# Ascending colon cecal junction carcinoma with prostate metastasis: A case report and literature review

**DOI:** 10.1097/MD.0000000000033308

**Published:** 2023-03-17

**Authors:** Wanshan Zhu, Jiaming Wu, Lexia Wu, Jincheng Meng, Cantu Fang, Huatang Zhang

**Affiliations:** a Zhongshan Hospital of Traditional Chinese Medicine Affiliated to Guangzhou University of Traditional Chinese Medicine, Zhongshan, China.

**Keywords:** cancer metastasis, colon carcinoma, metastatic carcinoma of the prostate

## Abstract

Colon carcinoma is the most common type of cancer, and a leading cause of cancer-related death. Clinically, the most common sites of metastases from colon carcinoma are the liver, lungs, peritoneum, and lymph nodes, while the incidence of metastases to the prostate is low. There are few relevant studies on colon carcinoma, most of them being case reports.

**Patient concerns:**

A 76-year-old man treated with radical resection of right colon carcinoma due to primary poorly differentiated adenocarcinoma of the cecum. Postoperative pathological examination suggested that he had cancer at the junction of the ascending colon and the cecum. He had received adjuvant chemotherapy after surgery. One year later, he received transurethral plasma resection of the prostate due to urinary system discomfort. Postoperative pathological immunohistochemistry suggested prostate metastasis of colorectal carcinoma, and he received individualized treatment, but this produced no clear survival benefit.

**Diagnoses:**

Ascending colon cecal junction carcinoma with prostate metastasis.

**Interventions:**

Radical resection, chemotherapy, anti-androgen therapy, surgery to relieve primary lesion obstruction symptoms, and local radiotherapy of the prostate.

**Outcomes:**

At present, clinical cases of colon carcinoma with prostate metastasis are rare. By sharing a rare case of ascending colon cecal junction carcinoma with prostate metastasis and reviewing the relevant literature, this paper explores and optimizes the clinical treatment of colon carcinoma with prostate metastasis.

## 1. Introduction

The incidence and mortality rate of male colorectal carcinoma is third in the world among cancers.^[1]^ With continual improvement of clinical diagnosis and medical technology, colon carcinoma patients survival has been significantly prolonged. However, the probability of cancer metastasis has also increased significantly, and the likelihood of a secondary cancer occurring as a metastasis involving the prostate gradually increases as well. Most prostatic cancers are primary cancers, and there have been few clinical reports or literature reports about the metastatic carcinoma of the prostate, especially metastasis from the colon.^[2]^ Therefore, selecting a treatment for these patients still depends primarily on relevant literature and clinical experience, as well as carefully chosen personalized programs that may respond to specific treatments to improve treatment efficiency.

### 1.1. Case report

**Figure 1.**
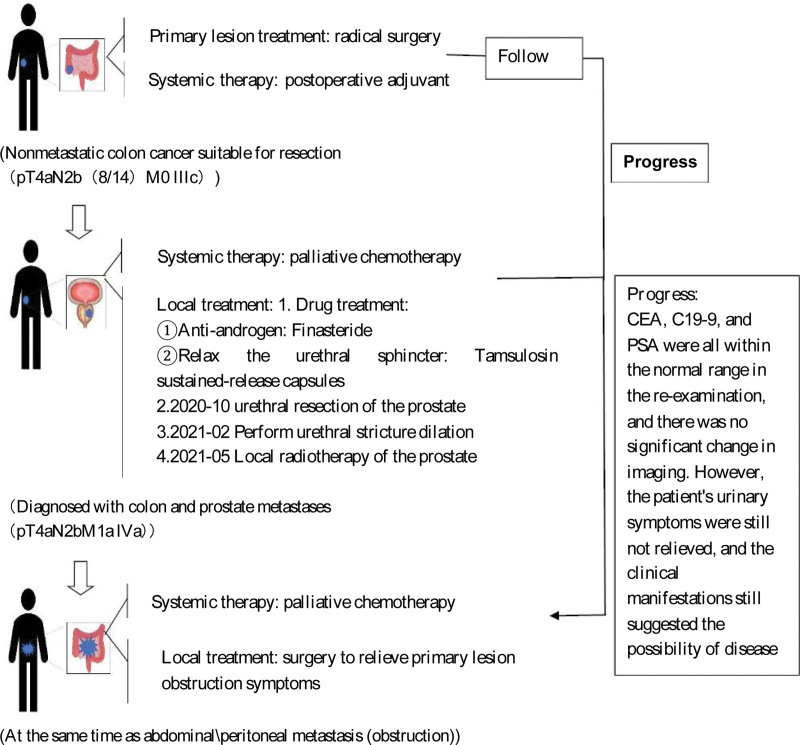
Timeline.

A 76-year-old man with no significant past medical or family history was referred to our hospital in July 2019 due to abdominal pain without obvious inducement. It was focused around the umbilical cord, caused paroxysmal exacerbation, and was accompanied by abdominal distension. Colonoscopy showed a cecal mass; pathological immunohistochemistry showed poorly differentiated adenocarcinoma (signet ring cell carcinoma) (Fig. 1). After excluding surgical contraindications, we performed laparoscopic radical resection of the right colon carcinoma. The postoperative pathological diagnosis was: ascending colon cecal junction carcinoma (moderate-poorly differentiated adenocarcinoma pT4aN2b (8/14) M0 p-stage IIIc). We performed postoperative adjuvant chemotherapy from August 2019 to February 2020. After that, the patient was rechecked regularly in our hospital, and there was no sign of cancer recurrence.

**Figure 2.**
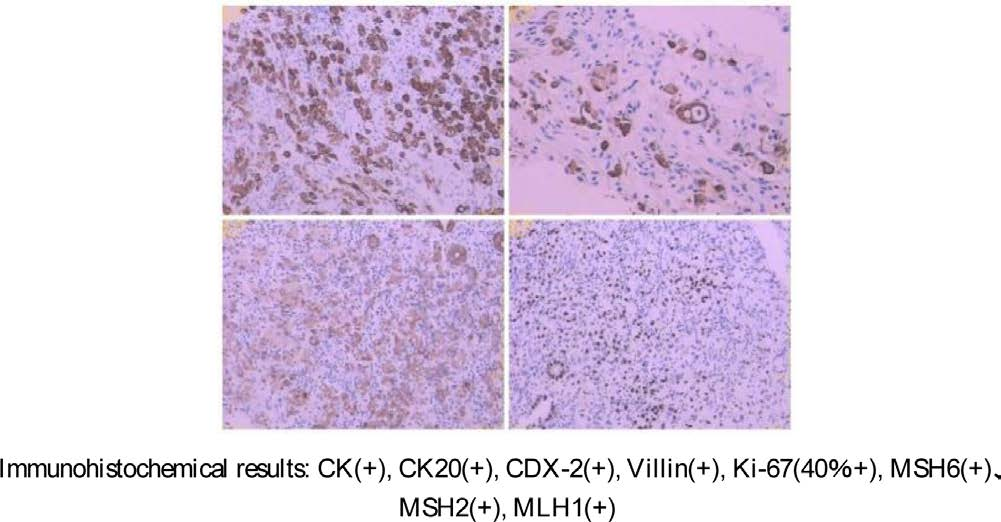
Immunohistochemistry after radical resection of the colon.

In August 2020, the patient experienced symptoms of delayed urination, shortened urinary line and a sense of frequent urination urgency. A color Doppler ultrasound showed no enlargement of the prostate, regular edge, complete capsule, uneven distribution of light spots, and strong light spots. Urodynamics showed bladder outlet obstruction IV (moderate and severe obstruction). Computed tomography (CT) showed that there was no obvious abnormality in the small calcification of the prostate, bladder or bilateral ureteral pelvic segments. Prostate specific antigen (PSA), carcinoembryonic antigen, carbohydrate antigen 19–9 and other related cancer indicators were within the normal range. Because we originally thought it was “benign prostatic hyperplasia,” we performed transurethral plasma resection of the prostate. The postoperative pathology showed: (prostate) adenocarcinoma, part of the cell morphology showed signet-ring differentiation, combined with medical history, and immunohistochemistry, tendency to metastasize from colon carcinoma to the prostate, cancer in vessels and cancer invasion in nerve fasciculus (Fig. 2). The patient was diagnosed with ascending colon cecal junction carcinoma with prostate metastasis. The stage was pT4aN2bM1a (IVA). Considering the clear source of colon carcinoma metastasis, we began palliative chemotherapy in December 2020, and local radiotherapy of the prostate in May 2021.

**Figure 3.**
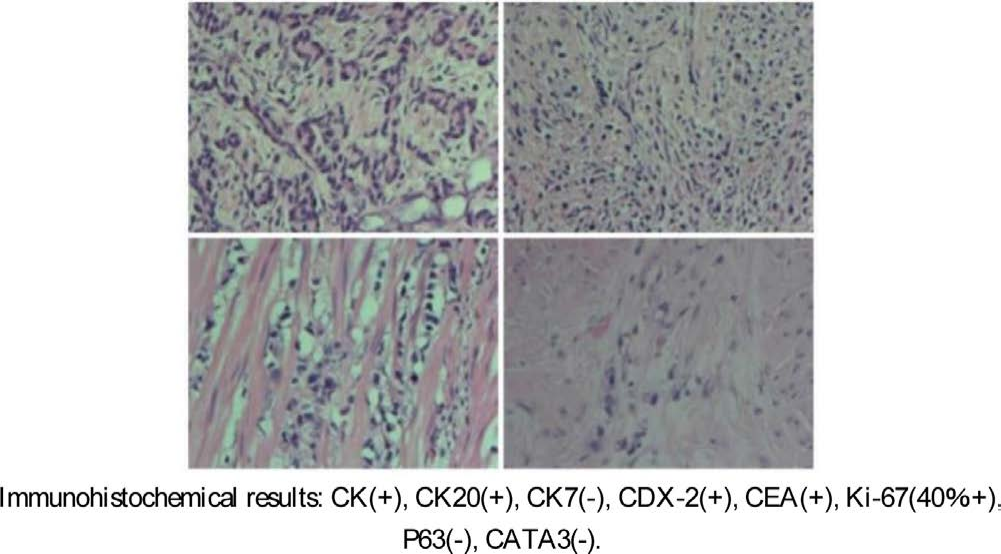
Immunohistochemistry after prostate surgery.

In July 2021, the patient’s abdominal pain was aggravated due to abdominal distension. An abdominal X-ray revealed changes in the incomplete intestinal obstruction. Thus, we performed catheter placement; the symptoms were relieved, and the patient was discharged from the hospital. In August 2021, the patient’s abdominal pain and abdominal distension were aggravated. An X-ray examination of the abdomen suggested that there may have been changes in the incomplete intestinal obstruction. Thus, we performed a catheterization for intestinal obstruction. The patient was discharged from the hospital after the symptoms were relieved. In August 2021, the patient experienced the same symptoms. CT examination showed a change in the incomplete obstruction, which may have been caused by the right lower lung being adherent to the small intestine. Laparoscopic exploration revealed a large amount of ascites in the abdominal cavity. Therefore, we were concerned that there may have been extensive planting and metastasis of the cancer. On September 10, 2021, the patient died due to the progression of the disease (Fig. 3).

## 2. Discussion

### 2.1. Pathogenesis and metastasis pathway

At present, there is no clear physiological or pathological etiology leading to this form of prostatic metastases after radical resection of colon carcinoma. The occurrence is rare, and the prognosis is poor, and multiple metastasis mechanisms are involved. To an extent, the shape and growth pattern of colon carcinoma can reflect the biological characteristics of the carcinoma.^[3]^ Postoperative pathology indicated that the cancer had invaded the serous layer (T4a). When the cancer invaded the visceral peritoneum, the cancer was able to invade the capillary venules, resulting in hematogenous metastasis. Cancer cells could also have been induced into circulation by extrusion of the cancer during surgery, or even strong peristalsis during obstruction. Systemic and local inflammatory reactions caused after surgery may also have accelerated the development of residual and exfoliated cancer cells, and stimulated the formation of new metastases.^[4]^ Furthermore, changes in anatomical structure may also have led to changes in cancer biology. The hard and dense Denonvilliers fascia structure between the prostate and colorectal region makes it difficult for colon carcinoma cells to penetrate and metastasize into the prostate. However, colon segment reanastomosis may cause a rupture of the Denonvillier fascia after resection of the primary lesion during colon carcinoma surgery. This makes the recurrence of colon cancer more likely to spread locally to the prostate.^[5]^ In addition, cancer can infiltrate directly or diffuse indirectly to periarterial structure and viscera. Surgery on colon carcinoma can cause peritoneal metastasis, and the cancer can spread along the surface of the peritoneal membrane, ligaments or mesangial cells, reaching the rectovesical pouch, finally landing in the prostate which is situated in the lower part of the bladder.

### 2.2. Clinical diagnosis

Accurate diagnosis of the disease affects the choice of treatment plan, and its therapeutic benefit and prognosis are also affected by it. Therefore, accurate diagnosis is crucial. The prostate is not a common site of secondary tumors, as most of the tumors in this region are primary tumors which are rarely metastatic prostate cancer. Therefore, the possibility of misdiagnosis and missed diagnosis is elevated.

The possibility of prostate metastasis should also be excluded. In elderly males with a history of cancer, urinary symptoms such as frequency of urination, urgency of urination, lack of urination or dysuria should be excluded as potentially indicative of prostate metastasis. Additionally, prostate space-occupying foci revealed by B-mode ultrasound, CT or other imaging examinations, with low or mildly elevated PSA and other related prostate tumor markers, with clinical symptoms consistent with or contradictory to auxiliary examination, should be excluded as well.

Similar morphological features are limited in determining the origin of cancers. The morphological characteristics of prostate cancer and adenocarcinoma of the colon show high similarity. Therefore, immunohistochemical and molecular diagnostic markers are important in determining the origin of primary malignancies and rare metastatic cancers. CDX2, CK20, β-catenin, PSA, P501S, and P504S may help to further differentiate prostate carcinoma from colon carcinomas in immunohistochemistry of poorly differentiated cancer involving the colorectal-prostatic region.^[6,7]^ (Table 1)

**Table 1:** Immunohistochemical expression of prostate cancer and colorectal cancer.

	PCa	CRCa
CDX2	0%	60%
CK20	10%	80%
β-catenin	0%	100%
PSA	80%	0%
P510S	80%	0%

CRCa = colon rectal carcinoma, PCa = prostatic carcinoma, PSA = prostate specific antigen.

However, secondary cancers may also lack unique pathological and immunohistochemical characteristics.^[7]^ This makes it difficult to arrive at an appropriate diagnosis. Therefore, understanding the patient’s relevant medical history and clinical background is also critical in these cases. In sum, patients with a history of colon carcinoma should be considered at risk of metastasis and diffusion of the colon, and therefore this should be excluded. Moreover, pathological and immunohistochemistry should also be improved to avoid possible unnecessary treatment.

### 2.3. Treatment and prognosis

For patients with advanced malignant tumors, personalized and multi-means comprehensive treatment can prolong survival times and improve quality of life. After completing the pathological and immunohistochemical examination, the patient was diagnosed with ascending colon cecal junction carcinoma with prostate metastasis (pT4aN2bM1a, p-stage IVA). In fact, the dissemination mechanism of colon carcinoma cells in this patient may have occurred in the early stages of the disease. Cancer cells could have entered the microenvironment of the prostate, which is a niche for them to be placed and controlled regarding their dormancy.^[8]^ Early or cryptic metastatic foci often have no obvious local symptoms or imaging signs. Therefore, the metastatic foci may have been overlooked, which would have mitigated the benefit of the patient’s treatment and lowered prognosis.

When colon carcinoma has distant metastasis, its prognosis is poor. Although distant metastasis is one of the reasons for the poor prognosis of cancer patients, according to relevant literature and reports, most deaths due to colon carcinoma are caused by the progression of primary foci. Therefore, primary foci are still the focus of treatment. This refers primarily to the systematic treatment of primary cancer, combined with the specific situation, and supplemented by the local treatment of metastatic foci. However, there is relevant clinical evidence that patients with colon carcinoma can benefit from local treatment of oligometastatic foci,^[9]^ including surgical resection and nonresectable local treatment such as ablation or radiotherapy. Yet, there is still no clear clinical evidence that patients’ progress can be prospectively predicted. We also cannot predict whether local treatment of these metastases will provide a significant survival benefit based on the clinical features, biomarkers or imaging examinations.

In sum, with the extension of cancer patients’ survival time, the incidence of colon carcinoma metastasis increases, and attention has gradually been drawn to improving its diagnosis. Prostate metastasis in colon carcinoma is rare and happens in the advanced stages of the cancer. Its prognosis is poor, and it is often misdiagnosed and overlooked in clinical examinations. Therefore, it is still necessary to improve the diagnosis rate for prostate metastasis. For patients with advanced unresectable metastatic colon carcinoma, the treatment is focused on improving patient quality of life. Therefore, it is appropriate to choose an individualized treatment scheme according to the patient’s condition, combined with chemotherapy, immunotherapy, targeted therapy, local radiotherapy or surgery.

## Acknowledgments

The researchers received no grants from funding agencies in either the public, commercial, or not-for-profit sectors, for this research.

## Author contributions

**Conceptualization:** Jincheng Meng, Cantu Fang.

**Data curation:** Jiaming Wu, Lexia Wu, Cantu Fang.

**Formal analysis:** Jiaming Wu, Lexia Wu.

**Investigation:** Huatang Zhang.

**Methodology:** Wanshan Zhu, Huatang Zhang.

**Supervision:** Jincheng Meng, Cantu Fang.

**Validation:** Jincheng Meng.

**Writing – original draft:** Wanshan Zhu, Huatang Zhang.

**Writing – review & editing:** Jiaming Wu, Lexia Wu.

## References

[1] Siegel RL Miller KD Fuchs HE. Cancer statistics, 2022. CA Cancer J Clin. 2022;72:7–33. 35020204 10.3322/caac.21708

[2] Wang JY. Prostate Disease Theory and Clinical Practice. Beijing Press; 2002;2:253–255.

[3] Liang J Wang D Pan Z. Multivariate analysis of recurrence and metastasis after radical resection of colorectal cancer. Cancer. 2004:564–7.

[4] Tohme S Simmons RL Tsung A. Surgery for cancer: a trigger for metastases. Cancer Res. 2017;77:1548–52. 28330928 10.1158/0008-5472.CAN-16-1536 PMC5380551

[5] Osunkoya AO Netto GJ Epstein JI. Colorectal adenocarcinoma involving the prostate: report of 9 cases. Hum Pathol. 2007;38:1836–41. 17868775 10.1016/j.humpath.2007.04.021

[6] Owens CL Epstein JI Netto GJ. Distinguishing prostatic from colorectal adenocarcinoma on biopsy samples: the role of morphology and immunohistochemistry. Arch Pathol Lab Med. 2007;131:599–603. 17425391 10.5858/2007-131-599-DPFCAO

[7] Bates AW Baithun SI. Secondary solid neoplasms of the prostate: a clinico-pathological series of 51 cases. Virchows Arch. 2002;440:392–6. 11956820 10.1007/s004280100505

[8] Kang A Lee JH Lin E. Metastatic colon carcinoma to the prostate gland. J Comput Assist Tomogr. 2013;37:463–5. 23674022 10.1097/RCT.0b013e3182898248

[9] Massaut E Bohlok A Lucidi V. The concept of oligometastases in colorectal cancer: from the clinical evidences to new therapeutic strategies. Curr Opin Oncol. 2018;30:262–8. 29746284 10.1097/CCO.0000000000000453

